# Snake Venom Cathelicidins as Natural Antimicrobial Peptides

**DOI:** 10.3389/fphar.2019.01415

**Published:** 2019-11-29

**Authors:** Elizângela de Barros, Regina M. Gonçalves, Marlon H. Cardoso, Nuno C. Santos, Octávio L. Franco, Elizabete S. Cândido

**Affiliations:** ^1^ Programa de Pós-Graduação em Biotecnologia, Universidade Católica Dom Bosco, Campo Grande, Brazil; ^2^ S-Inova Biotech, Universidade Católica Dom Bosco, Campo Grande, Brazil; ^3^ Centro de Análises Proteômicas e Bioquímicas, Programa de Pós-Graduação em Ciências Genômicas e Biotecnologia, Universidade Católica de Brasília, Brasília, Brazil; ^4^ Instituto de Medicina Molecular, Faculdade de Medicina, Universidade de Lisboa, Lisbon, Portugal

**Keywords:** natural peptides, host-defense peptides, cathelicidins, antimicrobial peptides, snake venom

## Abstract

Bioactive small molecules isolated from animals, plants, fungi and bacteria, including natural antimicrobial peptides, have shown great therapeutic potential worldwide. Among these peptides, snake venom cathelicidins are being widely exploited, because the variation in the composition of the venom reflects a range of biological activities that may be of biotechnological interest. Cathelicidins are short, cationic, and amphipathic molecules. They play an important role in host defense against microbial infections. We are currently facing a strong limitation on pharmacological interventions for infection control, which has become increasingly complex due to the lack of effective therapeutic options. In this review, we will focus on natural snake venom cathelicidins as promising candidates for the development of new antibacterial agents to fight antibiotic-resistant bacteria. We will highlight their antibacterial and antibiofilm activities, mechanism of action, and modulation of the innate immune response.

## Introduction

The rapid increase in microbial resistance to different drugs has been raising concerns worldwide, thus encouraging the search for effective alternative treatments (Nathan and Cars, 2014), especially when dealing with multidrug-resistant pathogens (Koo and Seo, 2019). This resistance has been associated with morbidity and mortality, not only in humans but also in animals, whereas the excessive and inappropriate use of antibiotics has been considered the main cause of bacterial resistance emergence (Sala et al., 2018; Koo and Seo, 2019).

For decades, there has been a lack of innovation in antibiotic classes, with these molecules being just optimized, and almost no new classes have been introduced on the market (Singh and Abraham, 2014). Given this problem, the development of new antimicrobial drugs is of great importance (Koo and Seo, 2019). Therefore, antimicrobial peptides (AMPs) represent potent and efficient candidates for the development of a new drug generation (Singh and Abraham, 2014). As a result, some AMPs have already reached clinical trial levels, and currently some are already available in the market, such as polymyxins B and E, bacitracins, and gramicidins (Mahlapuu et al., 2016; Costa et al., 2019).

AMPs are low molecular weight compounds, ranging from 12 to 50 amino acid residues (Forde and Devocelle, 2015). They can be naturally occurring, usually cationic and hydrophobic (Haney et al., 2019), are encoded by genes that remain conserved in the genome during evolution, and can be isolated from virtually all forms of living organisms (Mahlapuu et al., 2016).

Currently, AMPs can be produced on a large scale by chemical synthesis or by biotechnological methods such as recombinant expression in bacteria, yeast, plants, and others (Boto et al., 2018). The AMP production costs vary according to molecular size and complexity. Thus, biotechnological methods, as already mentioned, are being increasingly well developed, allowing greater production cost reduction when compared to chemical methods (Bommarius et al., 2010; Pachón-Ibáñez et al., 2017).

AMPs also have a broad spectrum of action, sometimes showing synergistic effects for their multiple modes of action (Boto et al., 2018). However, despite the great advantages, these interesting molecules could be relatively unstable, may have low specificity, with short plasma half-lives because of proteolytic degradation and, consequently, may have low solubility and oral bioavailability (Boto et al., 2018; Lau and Dunn, 2018). In addition, studies have shown that some *Escherichia coli* strains are already resistant to AMPs (Maria-Neto et al., 2012; Cardoso et al., 2017). Due to these disadvantages, researchers are increasingly striving to reach peptide optimization in order to increase their effectiveness. Even so, AMPs remain promising alternatives to conventional antibiotics (Mishra et al., 2017).

Some AMPs exhibit cytotoxicity against cancer cells and may also present immunomodulatory activity, acting indirectly in pathogen clearance (Yeung et al., 2011; Mahlapuu et al., 2016). This range of biological activities gives AMPs a high therapeutic potential, and this may be related to the fact that they exhibit different molecular structures (Mahlapuu et al., 2016). Diverse natural AMPs have many cysteine residues in their primary sequence, which favor stability for the formation of disulfide bonds (Salas et al., 2015).

The focus of this review is a class of cationic antimicrobial defense peptides, the cathelicidins, which are found in vertebrates and are encoded by innate immunity genes (Wei et al., 2015). Cathelicidins are phylogenetically old molecules, dating back at least 400 million years (Falcao et al., 2014). The first identification of cathelicidins was reported in mammalian bone marrow myeloid cells; therefore, these molecules were also named myeloid AMPs (Kościuczuk et al., 2012). Later, they were also found in bovine epithelial cells and neutrophils, including humans (Kościuczuk et al., 2012).

Cathelicidins can be found in the most diverse animal species, including mammals, fish, birds, amphibians, and reptiles (Wang et al., 2008). The snake venom comprises a rich biochemical source of bioactive molecules with varied structures and functions (Almeida et al., 2019), which provides a range of therapeutic effects with pharmacological and biotechnological applications (Almeida et al., 2018). Thus, snake venoms are explored in the search for natural molecules, such as the venom cathelicidin-related antimicrobial peptides (CRAMPs) of snakes. These molecules have high relevance at bacterial infections scenario, serving as a model for the design of new pharmaceuticals (Samy et al., 2011; Almeida et al., 2018; Almeida et al., 2019).

Cathelicidins from snake venom have been widely studied, mainly in the Elapidae and Viperidae families, a process that was greatly facilitated by the sequencing of the complete genome of these animals, enabling the identification of cathelicidins (van Hoek et al., 2014). Cathelicidins are multifunctional bioactive molecules derived from proteolytic cleavage. They were characterized by a gene-encoded signal peptide in the N-terminal segment and a highly conserved cathelin (pro-peptide) domain derived from the cathepsin L Inhibitor, followed by a structurally diverse C-terminal antimicrobial domain (mature peptide) (Zanetti et al., 1995; Gao et al., 2015). The amphipathic α-helical conformation is prevalent in cathelicidins, despite having hypervariable active peptide sequences among different species (Xhindoli et al., 2016).

In this context, this review provides information on snake venom AMPs, including their characterization, biological activities (with a core focus on the antibacterial and antibiofilm effects), modes of action, and structural profile.

### Snake Venom Cathelicidin-Related Antimicrobial Peptides

In the Reptilia class, the cathelicidin-related antimicrobial peptides (CRAMPs) from natural snake venom (**Table 1**) constitute an important family involved in the immune system defense, presenting not only antibacterial but also immunomodulatory properties (Bals et al., 2003; Kościuczuk et al., 2012; Mansour et al., 2014; Coorens et al., 2015; van Harten et al., 2018).

**Table 1:** Snake venom CRAMPs deposited in the Antimicrobial Peptide Database (APD), access to mature peptide and UniProtKB for precursor peptide.

ID	Peptide name	Source organism	Active peptide sequence	Number of residues (aa)		Activity^*^	Secondary structure/method	Reference
				Prepro	Mature			
AP00897/B6S2X0	NA-CATH	Chinese cobra *Naja atra*	KRFKKFFKKLKNSVKKRAKKFFKKPKVIGVTFPF	191	34	Antibacterial (G+ and G‑), antibiofilm	α-helix/NMR	(Zhao et al., 2008) (Du et al., 2015)
AP00896/B6D434	BF-CATH	Banded krait *Bungarus fasciatus*	KRFKKFFRKLKKSVKKRAKEFFKKPRVIGVSIPF	191	34	Antibacterial (G+ and G‑)	α-helix/NMR	(Zhao et al., 2008)
AP00895/B6S2X2	OH-CATH	King cobra *Ophiophagus hannah*	KRFKKFFKKLKNSVKKRAKKFFKKPRVIGVSIPF	191	34	Antibacterial (G+ and G‑), enzyme inhibitor	α-helix/NMR	(Zhao et al., 2008)
AP01239/B6D434	Cathelicidin-BF	Banded krait *B. fasciatus*	KFFRKLKKSVKKRAKEFFKKPRVIGVSIPF	191	30	Antibacterial (G+ and G‑), enzyme inhibitor antifungal, antitumor	α-helix/NMR	(Wang et al., 2008)
Not available**/**U5KJJ1	Pt_CRAMP1	Eastern-brown-snake *Pseudonaja textilis*	KRFKKFFMKLKKSVKKRVMKFFKKPMVIGVTFPF	184	34	Antibacterial (G+ and G‑)	α-helix/NMR	(Falcao et al., 2014)
Not available/U5KJM6	Pt_CRAMP2	Eastern-brown-snake *P. textilis*	KRFKKFFRKLKKSVKKRVKKFFKKPRVIGVTIPF	184	34	Antibacterial (G+ and G‑)	α-helix/NMR	(Falcao et al., 2014)
AP02424/U5KJM4	Crotalicidin (Ctn)	South American rattlesnake *Crotalus durissus terrificus*	KRFKKFFKKVKKSVKKRLKKIFKKPMVIGVTIPF	194	34	Antibacterial (G+ and G‑), antifungal, antitumor	α-helix/NMR	(Falcao et al., 2014) (Falcao et al., 2015)
AP02423/U5KJC9	Batroxicidin (BatxC)	South American pit vipers *Bothrops atrox*	KRFKKFFKKLKNSVKKRVKKFFRKPRVIGVTFPF	189	34	Antibacterial (G+ and G‑), antiparasitic	Unknown	(Falcao et al., 2014)
Associated with crotalicidin/U5KJT7	Lutzicidin	South American pit vipers *Bothrops lutzi*	KRFKKFFKKLKNNVKKRVKKFFRKPRVIGVTIPF	189	34	Antibacterial (G+ and G‑)	Unknown	(Falcao et al., 2014)
Associated with crotalicidin/U5KJZ2	Lachesicidin	South American pit vipers *Lachesis muta rhombeata*	KRFKKFFKKVKKSVKKRLKKIFKKPMVIGVTFPF	194	34	Antibacterial (G+ and G‑)	Unknown	(Falcao et al., 2014)
AP02569/A0A0G3DRW6	Hc-CATH	Blue-banded sea snake	KFFKRLLKSVRRAVKKFRKKPRLIGLSTLL	187	30	Antibacterial (G+ and G‑), antifungal	α-helix/CD	(Wei et al., 2015)
		*Hydrophis cyanocinctus*						
AP02964/A0A2P1AGD5	CATHPb1	Burmese python *Python bivittatus*	KRFKKFFRKIKKGFRKIFKKTKIFIGGTIPI	175	31	Antibacterial (G+ and G‑), anti-MRSA and VRSA, antibiofilm, antifungal	α-helix/CD	(Cai et al., 2018)
AP03077/A0A4D6DT23	SA-CATH	Chinese snake *Sinonatrix annularis*	KFFKKLKKSVKKHVKKFFKKPKVIGVSIPF	191	30	Antibacterial (G+ and G‑), antibiofilm, antifungal, anti-inflammatory	Unknown	(Wang et al., 2019)

(*) Activity abbreviations: inhibiting both Gram-positive and Gram-negative bacteria (G+, G‑); methicillin-resistant and vancomycin-resistant S. aureus (MRSA and VRSA, respectively).

The first discovery of reptile CRAMPs was reported in 2008 (Zhao et al., 2008). These authors described peptides isolated from venom and tissues of the three Asian elapid species (**Table 1**). The NA-CATH peptide was identified from *Naja atra* (Chinese cobra), OH-CATH from *Ophiophagus hannah* (king cobra), and BF-CATH (Zhao et al., 2008) and cathelicidin-BF (Wang et al., 2008) from *Bungarus fasciatus* (Banded krait), all from constructed snake venom gland cDNA libraries. The precursor peptide of BF-CATH and cathelicidin-BF is composed of 191 amino acid residues with a conserved cathelin domain that, by proteolytic cleavage, results in two 34- and 30-amino-acid active peptides, respectively (Wang et al., 2008; Zhao et al., 2008). Falcao et al. (2014) identified two cathelicidin peptide precursors from venom gland cDNA libraries of an elapid snake, *Pseudonaja textilis* (Eastern brown snake), named Pt_CRAMP1 and Pt_CRAMP2. In addition, other CRAMPs from snake venom have been identified and characterized by Falcao et al. (2014) (**Table 1**). These peptides were isolated from four different species of South American pit-vipers (rattlesnakes and jararacas), including *Crotalus durissus terrificus* [crotalicidin (Ctn)], *Bothrops atrox* [batroxicidin (BatxC)], *Bothrops lutzi* (lutzicidin), and *Lachesis muta rhombeata* (lachesicidin). All these molecules are classified as vipericidins (Falcao et al., 2014).

An elapidic cathelicidin was identified by Wei et al. (2015) and named Hc-CATH (**Table 1**). The authors isolated this molecule from the sea snake *Hydrophis cyanocinctus* (Blue-banded sea snake). Furthermore, the expression of the native peptide was confirmed in the animal venom gland, spleen, lung, and skin, the last one with the lowest level of expression (Wei et al., 2015). Lastly, one more cathelicidin, CATHPb1, was reported by Cai et al. (2018), isolated from the Burmese python *Python bivittatus*, native to southeast and southwest Asia and depicted as among the five largest snake species in the world (Cai et al., 2018).

Overall, in both groups of vipericidins and elapids, the common physicochemical features are extremely preserved sequences among the vertebrate cathelicidin precursors. These molecules present a mature or active peptide with 30- to 34-amino acid residues and an amphipathic α-helical structural profile. Additionally, techniques such as circular dichroism (CD) (Falcao et al., 2015; Wei et al., 2015; Cai et al., 2018) and nuclear magnetic resonance (NMR) (Du et al., 2015) spectroscopies have been used to elucidate and confirm the predicted secondary structures, as specified in **Table 1** (Wang et al., 2008).

Recently, Wang et al. (2019) reported a new cathelicidin (SA-CATH), identified, and characterized for the first time, from the Chinese snake *Sinonatrix annularis*, which belongs to the Colubridae family (**Table 1**). SA-CATH is composed of 30 amino acid residues, and presents high sequence similarity with other cathelicidins from the Elapidae and Viperidae snake families (Wang et al., 2019).

Besides the natural peptides described herein, it is important to note that computational strategies, an attractive model for designing novel AMPs, have been developed to generate increasingly specific analogs with improved physicochemical characteristics (Chen et al., 2011; Blower et al., 2015; Kim et al., 2017; Júnior et al., 2018).

Apart from the snake venom-derived cathelicidins cited above, recent studies have also reported cathelicidin-like peptides in turtles (Qiao et al., 2019; Shi et al., 2019) and crocodiles (Barksdale et al., 2017; Chen et al., 2017) (**Supplementary Table 1**). Overall, only a few reptilian cathelicidins have been identified so far, with a higher prevalence of snakes venoms.

### Antibacterial and Antibiofilm Activity of Snake Venom Cathelicidins

As previously mentioned, bacterial resistance to antibiotics has become a worldwide public health problem (Sultan et al., 2018). Another critical problem is pictured by bacterial biofilms, which represent an adaptive resistance that difficult bacteria-related diseases treatment (Ciofu and Tolker-Nielsen, 2019). In addition, the clinical relevance of biofilms has been constantly emphasized, as they account for ~65% of all human infections (Haney et al., 2018). Thus, cathelicidins derived from snake venoms are interesting alternatives to counter bacterial pathogens. As shown in **Table 1**, 11 natural cathelicidins have had their antibacterial activity reported against Gram-positive and -negative bacteria. Regarding the antibiofilm activity, only two natural cathelicidins were explored.

Studies performed by Dean et al. (2011); Blower et al. (2015), and Du et al. (2015) reported the antibacterial and antibiofilm potential of cathelicidin NA-CATH. Dean et al. (2011) reviewed the antibacterial and antibiofilm effect of NA-CATH against *Staphylococcus aureus* (**Table 2**). No hemolytic activity of this peptide against horse erythrocytes was observed (**Table 2**) (Dean et al., 2011). Later, Blower et al. (2015) also examined the antibacterial and antibiofilm activity of this same molecule, but against *Burkholderia thailandensis* (**Table 2**). The ability of NA-CATH to eradicate preformed biofilms of *B. thailandensis* was also assessed; however, the peptide showed no such effect (Blower et al., 2015). Still exploring the NA-CATH peptide, Du et al. (2015) evaluated the determinants for antibacterial activity through solution NMR experiments using lipid vesicles (liposomes) and fluorescence quenching, mimicking the activity on the bacterial membrane.

**Table 2:** Antibacterial, antibiofilm, and cytotoxic activity of snake venom cathelicidins against standard and clinical isolate bacterial strains, including multidrug-resistant Gram-positive and -negative bacteria.

Peptide name	Bacterial strains	Activity (µg.mL^-1^)			Reference
		Antibacterial	Antibiofilm	Cytotoxic	
NA-CATH	*Burkholderia thailandensis*	3.6**	>3.6**	NT	(Blower et al., 2015)
	*Staphylococcus aureus*	2.9**	10**	>100 (horse erythrocytes)	(Dean et al., 2011)
Cathelicidin-BF	*Acinetobacter calcoaceticus*	2.3*	NT	>400 (murine macrophages RAW 264.7 and human hepatic tumor cells HepG2; human erythrocytes)	(Wang et al., 2008)
	*Bacillus cereus*	1.2*			
	*Bacillus pumilus*	9.4*			
	*Bacillus subtilis*	9.4*			
	*Enterococcus faecium*	150*			
	*Escherichia coli*	0.6–2.3*			
	*Klebsiella pneumoniae*	0.3–9.4*			
	*Pseudomonas aeruginosa*	1.2–18.7*			
	*Pseudomonas luteola*	1.2*			
	*Salmonella typhi*	1.2*			
	*Sacharibacillus kuerlensis*	4.7*			
	*Serratia marcescens*	>400*			
	*Sphingobacterium siyangense*	9.4*			
	*S. aureus*	4.7– > 400*			
OH-CATH	*Acinetobacter baumannii*	16*	NT	>415 (human erythrocytes)	(Zhao et al., 2008) (Zhang et al., 2010) (Falcao et al., 2014)
	*E. coli*	0.25–20*			
	*Enterobacter aerogenes*	2–4*			
	*Enterobacter cloacae*	1–8*			
	*Enterococcus faecalis*	64– > 128*			
	*K. pneumoniae*	8*			
	*P. aeruginosa*	0.5–16*			
	*S. aureus*	4–64*			
Pt_CRAMP1	*A. baumannii*	16*	NT	210 (human erythrocytes)	(Falcao et al., 2014)
	*E. faecalis*	32–64*			
	*E. coli*	2–16*			
	*K. pneumoniae*	32*			
	*P. aeruginosa*	8–32*			
	*S. aureus*	32–64*			
	*Streptococcus pyogenes*	16*			
Crotalicidin (Ctn)	*A. baumannii*	16*	NT	>415 (erythrocytes; macrophages RAW 264.7)	(Falcao et al., 2014) (Oliveira-Júnior et al., 2018) (Pérez-Peinado et al., 2018)
	*E. faecalis*	32–128*			
	*E. coli*	0.25–16*			
	*K. pneumoniae*	4–16*			
	*P. aeruginosa*	1–16*			
	*S. aureus*	32*			
	*S. pyogenes*	16*			
Batroxicidin (BatxC)	*A. baumannii*	16*	NT	>425 (erythrocytes; macrophages RAW 264.7)	(Falcao et al., 2014) (Oliveira-Júnior et al., 2018)
	*E. faecalis*	32–128*			
	*E. coli*	0.25–16*			
	*K. pneumoniae*	8–16*			
	*P. aeruginosa*	1–16*			
	*S. aureus*	32*			
	*S. pyogenes*	16*			
Hc-CATH	*A. baumannii*	>200*	NT	>200 (mouse macrophages of the normal cell line (L929), human liver tumor cells (HepG2), prostate cancer cells (PC3), and human erythrocytes)	(Wei et al., 2015)
	*B. cereus*	9.4*			
	*B. subtilis*	75*			
	*E. faecalis*	>200*			
	*E. faecium*	37.5*			
	*E. coli*	2.3–9.4*			
	*Klebsiella oxytoca*	4.7*			
	*K. pneumoniae*	4.7–75*			
	*Proteus mirabilis*	4.7*			
	*Proteus vulgaris*	>200*			
	*P. aeruginosa*	18.7– >200*			
	*Salmonella paratyphi*	4.7*			
	*S. marcescens*	>200*			
	*Shigella dysenteriae*	0.6*			
	*Stenotrophomonas maltophilia*	9.4– > 200*			
	*S. aureus*	4.7– > 200*			
	*Staphylococcus epidermidis*	>200*			
CATHPb1	*B. cereus*	1.17*	NT	>100 (human erythrocytes; normal human liver cells HL-7702 and mouse peritoneal macrophages MPMs).	(Cai et al., 2018)
	*Dysentery bacillus*	1.17*			
	*E. faecalis*	75*			
	*E. faecium*	9.38*			
	*E. coli*	9.38*	2.5** and 37.5***		
	*K. oxytoca*	75*	6.25** and 25***		
	*K. pneumoniae*	18.75*	NT		
	*Nocardia asteroids*	9.38*			
	*P. aeruginosa*	9.38–37.5*	6.25** and 44***		
	*S. paratyphi*	18.75*	NT		
	*S. aureus*	4.69–37.5*	7–11.8** and 30.5–55.3***		
	*S. epidermidis*	18.75*	NT		
	*S. maltophilia*	4.69*			
SA-CATH	*B. cereus*	4.69*	NT	>200 (human erythrocytes; human keratinocyte cell line HaCaT and mouse peritoneal macrophages MPMs)	(Wang et al., 2019)
	*B. subtilis*	18.75*			
	*E. faecium*	37.5*			
	*E. coli*	18.75–75*	40**		
	*K. pneumoniae*	37.5*	NT		
	*N. asteroids*	37.5*			
	*P. aeruginosa*	37.5*			
	*Shigella dysenteriae*	37.5*			
	*S. aureus*	75*			

*MIC, minimal inhibitory concentration; **IC50, minimal concentrations resulting in 50% of inhibition; and ***EC50, minimal concentrations resulting in 50% of eradicated preformed biofilms; NT, not tested.

According to Zhao et al. (2008), the BF-CATH peptide showed antibacterial activity against Gram-positive and -negative strains, as predicted in relation to the described activity of the OH-CATH peptide; however, further studies remain necessary to confirm this potential. In the same year, Wang et al. (2008) reported the antimicrobial activity of cathelicidin-BF against 40 standard strains and clinical isolates, including multidrug-resistant strains of microorganisms, compared to antibiotics for therapeutic use. Cathelicidin-BF was capable of effectively inhibiting and killing bacteria, especially Gram-negative strains (**Table 2**) and also showed inhibitory activity against some fungal species, including saprophytic ones. In addition, Wang et al. (2008) demonstrated that the presence of salts such as phosphate buffer and sodium chloride in solutions can improve the antibacterial activity of cathelicidin-BF compared to the water environment, against standard strains. Furthermore, hemolytic and cytotoxic properties were not reported in human erythrocytes and murine macrophages (RAW 264.7) and human hepatic tumor cells (HepG2), respectively, at the highest concentration tested (**Table 2**). Moreover, this peptide showed high serum stability for approximately 2 h in mice blood plasma (Wang et al., 2008).

For the Asian snake-derived cathelicidin OH-CATH, Zhao et al. (2008) and Zhang et al. (2010) have reported potent antibacterial activities. Zhao et al. (2008) tested OH-CATH peptide in the presence of 1% NaCl against eight bacterial strains (**Table 2**), inhibiting all strains and showing better results for a *Enterobacter cloacae* multidrug-resistant clinical isolate strain. However, in the study conducted by Zhang et al. (2010), the efficacy of the same molecule was evaluated and confirmed against the 11 standard and clinical isolates of bacterial strains (**Table 2**). The hemolytic activity has not been observed for OH-CATH in either of the studies described (**Table 2**).

The antimicrobial activity of peptides Pt_CRAMP1, Ctn, and BatxC and OH-CATH (used as the control) was analyzed by Falcao et al. (2014) against Gram-negative and -positive bacteria, presenting a better inhibition toward standard and clinical isolate strains of Gram-negative bacteria (**Table 2**). Therefore, Ctn and BatxC inhibited the clinical isolates of *Klebsiella pneumoniae*, resulting in similar data obtained for OH-CATH used in this same study as the reference. For these same strains, the elapid peptide Pt_CRAMP1 was less potent than the two vipericidins (Ctn and BatxC) and OH-CATH, although it presented minimal inhibitory concentrations (MICs) similar to other Gram-negative and -positive bacterial strains (**Table 2**). Furthermore, in comparison with these peptides mentioned, Pt_CRAMP1 showed the highest hemolytic activity, making it less selective (Falcao et al., 2014).

According to the antibacterial assays reported by Oliveira-Junior et al. (2018), both vipericidins (Ctn and BatxC) show activity toward standard and multidrug-resistant bacterial strains, having selectivity for clinically isolated Gram-negative bacterial strains (**Table 2**) without presenting hemolytic or cytotoxic effects (Oliveira-Júnior et al., 2018).

Addressing the antimicrobial potential of Ctn, Pérez-Peinado et al. (2018) reported the activity of Ctn against standard strains of *E. coli* and *Pseudomonas aeruginosa* (**Table 2**). In addition, this peptide showed a bactericidal effect against the same strains but at slightly higher concentrations (**Table 2**) (Pérez-Peinado et al., 2018).

Similarly to the *in vitro* studies described above, Wei et al. (2015) also reported the efficacy of a natural cathelicidin, derived from sea snake venom, Hc-CATH. In this study, the antimicrobial effect of this peptide was evaluated against human and marine pathogenic bacteria, besides fungal strains. Overall, the peptide showed potent inhibition toward standard and clinical isolates strains of Gram-negative and -positive bacteria. Moreover, Hc-CATH exhibited better MICs against *Shigella dysenteriae* followed by *E. coli* strains, among the 38 bacterial strains tested (**Table 2**). Furthermore, this peptide did not present hemolysis and cytotoxicity effects at the highest concentration tested, as shown in **Table 2** (Wei et al., 2015).

Cai et al. (2018) demonstrated that the CATHPb1 peptide has broad-spectrum antimicrobial activity *in vitro* against various microorganisms, including drug-resistant strains. When compared to antibiotics commonly used in the clinic, this showed more effectiveness and an excellent bactericidal potential in killing kinetic assays. According to Cai et al. (2018), CATHPb1 was also capable of inhibiting the formation of biofilms and eradicating preformed or mature biofilms (*E. coli*, *P. aeruginosa*, *Klebsiella oxytoca*, and *S. aureus*) (**Table 2**), and fungal strains (*Candida albicans* and *Candida glabrata*). Moreover, this potent peptide protected mice against the skin and systemic infection caused by methicillin-resistant and vancomycin-resistant *S. aureus*. In the *in vivo* model, this peptide increased the survival of mice with bacterial burden, without leading to the hemolysis of human erythrocytes (**Table 2**) (Cai et al., 2018).

Recently, Wang et al. (2019) evaluated the antimicrobial effects of SA-CATH against thirteen bacterial strains, including Gram-positive and -negative standard and clinical isolates (**Table 2**), as well as fungal strains, compared to antibiotics of clinical use. The best SA-CATH results were observed against *Bacillus cereus*, followed by *E. coli*. This peptide could completely inhibit and kill both bacterial strains within 30 min of peptide exposure. According to Wang et al. (2019), this peptide was also capable of inhibiting *E. coli* biofilm formation. Furthermore, hemolytic and cytotoxic activities were not reported against human erythrocytes and other mammalian cells, at the highest concentration tested (**Table 2**) (Wang et al., 2019). Considering the promising antibacterial and antibiofilm activities of cathelicidin peptides presented in this section, we can maintain the hypothesis that natural peptides from snake venoms are very rich sources of therapeutic agents that may be used for the treatment of multiresistant infectious diseases. However, further studies are still required to better evaluate the potential antibiofilm activity and the potential for the eradication of preformed biofilms of several of the cathelicidins described herein. Despite the scarce number of studies, we observed that the group of snakes is more explored than other reptiles. Antimicrobial properties of turtles and alligators cathelicidins have been reported (Barksdale et al., 2017; Chen et al., 2017; Qiao et al., 2019; Shi et al., 2019) (**Supplementary Table 1**). However, much information about cathelicidins reptile is scarce. Given this, further studies are needed to evaluate the antimicrobial potential of the various cathelicidins described here.

### Mechanisms of Antibacterial Activity of Snake Venom Cathelicidins

The mechanism of action of AMPs shows significant differences in the way it operates when compared to antibiotics. This is because they affect multiple targets, making it difficult to experience microbial resistance, unlike conventional antibiotics, which attack one specific target (Singh and Abraham, 2014; Mishra et al., 2018). One of the main mechanisms triggered by AMPs and cathelicidins is their binding to the target membrane, followed by its permeabilization and/or disruption, thus causing bacterial death (Mahlapuu et al., 2016). This membrane interaction may be receptor-mediated or not, where most vertebrate AMPs, including cathelicidins, do not specifically interact with receptors (Kumar et al., 2018). Thus, it can be seen that the mechanisms of action performed by the cathelicidins are the same as those exerted by most cationic AMPs, namely, the three main and best described bacterial membrane interaction models: carpet model, barrel model, and toroidal pore model (Agier et al., 2015).

**Figure 1:**
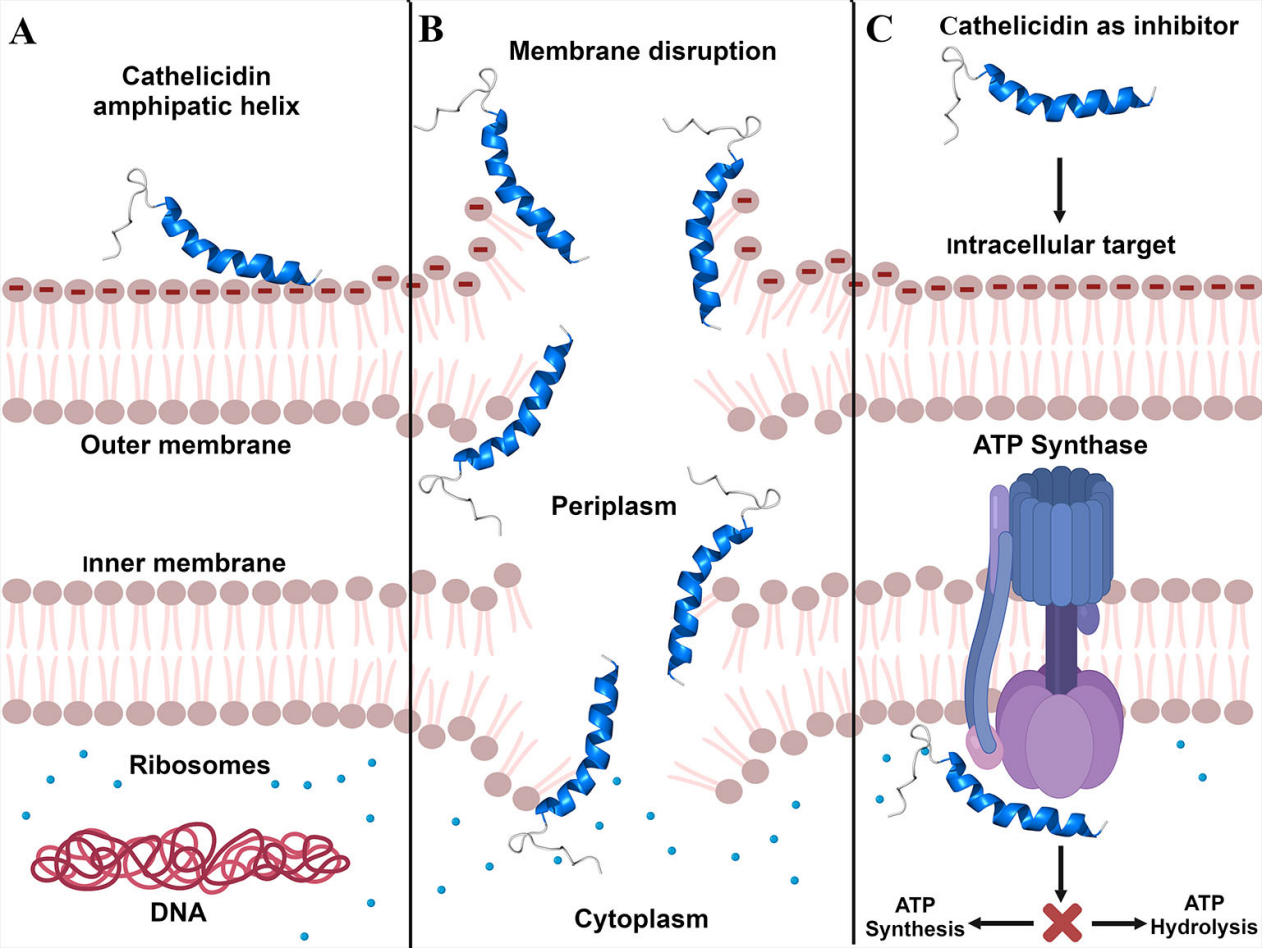
Cathelicidins' mechanisms of action. **(A)**, Cathelicidin structural modification by electrostatic interaction with Gram-negative bacterial membrane. **(B)**, Bacterial membrane disruption caused by cathelicidins. **(C)**, Inhibition of ATP synthesis/hydrolysis caused through cathelicidins binding to ATP synthase. Created with BioRender.com.

It is known that the cathelicidins are prone to be linear (Findlay et al., 2016) and form amphipathic α-helices upon coming into contact with bacterial membranes (**Figure 1A**). This feature plays an essential role in their activity (Blower et al., 2015), as the main broad-spectrum antimicrobial property of the cathelicidins is the interaction with the bacterial membrane (Pérez-Peinado et al., 2018). Studies conducted by Wang et al. (2011) and Wei et al. (2015) have already proven that cathelicidins are capable of causing the rupture of bacterial cell membrane, leading to the leakage of cytoplasmic content (**Figure 1B**) and, consequently, kill the bacteria. Wang et al. (2011) imaged by scanning electron microscopy (SEM) the bacterium *Propionibacterium acnes* treated with cathelicidin-BF and observed membrane rupture and cell disruption in 30 min. In that same year, Zhou et al. (2011) also evaluated cathelicidin-BF (or BF-30) by transmission electron microscopy. In that study, *P. aeruginosa* and *S. aureus* treated with BF-30 for 2 h had partial membrane rupture and intracellular content leakage (Zhou et al., 2011). In the study conducted by Wei et al. (2015), the interaction of Hc-CATH with *E. coli* and *S. aureus* was analyzed by flow cytometry, which showed a rapid binding of peptide to the bacterial cells, in about 5 min. In the same study, the deformation and rupture of the bacterial cells in 30 min was also shown, through SEM, with extravasation of the intracellular content (Wei et al., 2015). These data reported here suggest that cathelicidin-BF and Hc-CATH are potent peptides that act on bacterial membrane destruction, of both Gram-negative and -positive bacteria (Wang et al., 2011; Wei et al., 2015).

In addition to membrane targets, cathelicidins may also act intracellularly, binding or degrading molecules, including DNA, RNA, and proteins. They can interfere with DNA and RNA synthesis and impair the functions of enzymes and other proteins, which are crucial molecules for cell survival (Agier et al., 2015; van Harten et al., 2018). Still in the context of intracellular targets, it was observed that cathelicidins are capable of inhibiting ATP synthase (Azim et al., 2016), the biological nanomotor that generates about 95% energy of the ATP for the cells (Laughlin and Ahmad, 2010). The inhibition of this mechanism deprives cells of ATP, affecting cell development, which results in cell death (Azim et al., 2016).

To identify potential ATP synthase inhibitors (**Figure 1C**), Azim et al. (2016) tested the cathelicidin OH-CATH and other peptides on *E. coli*. The authors found that OH-CATH was capable of inhibiting approximately 90% of ATP synthase activity through a reversible non-covalent interaction. Studies by Laughlin and Ahmad (2010) and Ahmad et al. (2015) also investigated the inhibition of ATP synthase in *E. coli*, but with peptides from other classes (Laughlin and Ahmad, 2010; Ahmad et al., 2015; Azim et al., 2016). In view of this, we observed that there are few studies on the intracellular targets of cathelicidins, although a range of mechanisms has already been identified. Therefore, we emphasize the importance of investing in and deepening studies on cathelicidin molecular targets, since they are promising for the development of new drugs.

### Immune Modulation Triggered by Snake Venom CRAMPs

Cathelicidins were described mainly in the context of their antimicrobial activity; however, over the years, other biological functions have been uncovered and appreciated (Zanetti, 2005). In addition to direct bactericidal properties, cathelicidins can trigger specific defense responses, including immunomodulatory activities, as well as modulation of chemokine expression in pro- and anti-inflammatory responses. Furthermore, the promotion of wound healing and the inhibition of cellular apoptosis have also been proposed (Mansour et al., 2014; Coorens et al., 2015). These peptides are often stored in neutrophil and macrophage secretory granules and can be released extracellularly by leukocytes upon activation. In addition, their expression was also reported in keratinocytes or epithelial cells (Boman, 1995; Zanetti, 2005; Kościuczuk et al., 2012). Therefore, several actions of these host defense peptides (HDPs) or natural antibiotics are promoted by their direct interaction with other cells of the innate immune system, including monocytes and dendritic, epithelial, and T cells (Mansour et al., 2014), besides the activation of specific receptors (Gupta et al., 2015).

**Figure 2:**
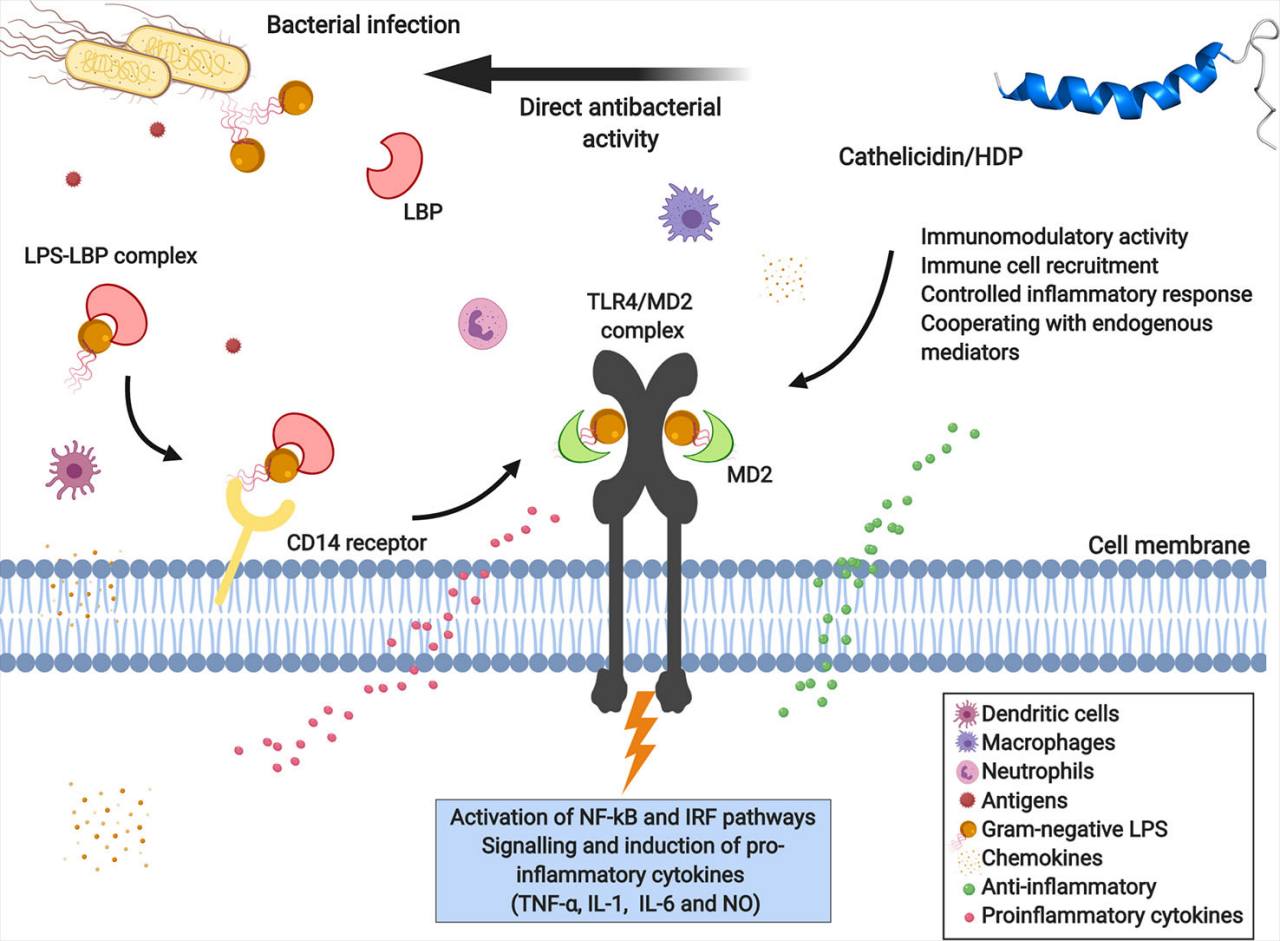
Immunomodulatory response of cathelicidins toward bacterial LPS mediated by TLR4-MD2 complex receptor signaling. Lipopolysaccharide (LPS), the main component of the outer membrane of Gram-negative bacteria, is recognized and activated by immune defense cells, and can bind to membrane receptors of variable specificity and induce the synthesis of inflammatory mediators. LPS is recognized by LBP (LPS-binding proteins) and the LPS-LBP complex binds to the CD14 receptor, a leukocyte membrane-expressed glycoprotein. In turn, LPS is presented to the TLR4-MD2 complex (Toll-like receptor 4 and myeloid differentiation factor 2), activating the transcription factors NF-κB (nuclear factor-κB) and IRF (interferon regulatory factors), consequently inducing the production of pro-inflammatory cytokines, chemokines, and nitric oxide (NO). The direct interaction of cathelicidin with bacterial LPS, the CD14 co-receptor or TLR4, allows modulation of the immune response, reducing its pro-inflammatory effects. Created with BioRender.com

The sea snake cathelicidin Hc-CATH exhibits potent antimicrobial and anti-inflammatory activity, inhibiting pro-inflammatory cytokines like tumor necrosis factor α (TNF-α), interleukins (IL-1 and IL-6), and nitric oxide (NO) production induced by lipopolysaccharide (LPS). Hc-CATH is capable of neutralizing LPS toxicity by direct binding to LPS molecule and Toll-like receptor 4 (TLR4) and myeloid differentiation factor 2 (MD2), which in turn inhibits LPS-induced inflammatory response pathways when bound to the TLR4/MD2 receptor complex, as shown in **Figure 2** (Wei et al., 2015).

Moreover, the peptide CATHPb1 also shows immuno-modulatory activity *in vivo* (Cai et al., 2018). This peptide can rapidly modulate and direct defense cells, like macrophages and neutrophils, to the site of infection with intense cell proliferation (**Figure 2**), thereby enhancing their bactericidal functions (Cai et al., 2018). Increased apoptosis of neutrophil-mediated bacteria was clearly observed by Cai et al. (2018), with CATHPb1 acting in synergy with cytokines or β-defensins. Besides, CATHPb1 promotes an improvement in chemokine levels and decreases the production of pro-inflammatory cytokines (**Figure 2**), without undesirable cytotoxicity (Agier et al., 2015).

Cathelicidin SA-CATH exhibited potent anti-inflammatory activity by inhibiting the production of LPS-induced proinflammatory cytokines (NO, TNF-α, and IL-6) in mouse peritoneal macrophage cells (MPMs) (Wang et al., 2019).

Finally, in the study by Coorens et al. (2017), it was observed that different cathelicidins predominantly show immunomodulatory functions by neutralizing LPS and lipoteichoic acid (LTA), as well as inhibiting macrophage activation. Besides, several cathelicidins increased chemokine expression by RAW 264.7 murine cells (Coorens et al., 2017). However, these authors confirm that these intrinsic properties may differ between the various cathelicidins and interspecies (Coorens et al., 2017).

### Snake Venom CRAMPs Structural Profile

Several natural AMPs have been isolated over the years and, according to their efficacy in inhibiting/killing pathogenic microorganisms, further studies have been performed to draw a structure/function relationship for these molecules (Cardoso et al., 2018b). For instance, extensive structural works have been performed with well-known natural AMPs, including magainin (Bechinger et al., 1993), indolicidin (Ladokhin et al., 1997), and LL-37 (Oren et al., 1999), among others. These studies have provided crucial information on AMP aggregation (Jean-Francois et al., 2008), pore formation (Hasan et al., 2018), membrane disruption and/or translocation (Ulmschneider, 2017), as well as possible interaction with intracellular targets (Zahn et al., 2014). Moreover, the understanding of natural AMP structure has enabled the structure-guided design of improved variants, which currently represents a large field of research in terms of antimicrobial agents (Torres et al., 2018). In the case of natural CRAMPs derived from snake venom, a few studies have reported how these peptides are organized at the structural level. Therefore, this section will focus on the main findings regarding the structural arrangements adopted by snake venom CRAMPs and how this has been related to their antimicrobial properties.

One of the first studies to investigate the secondary structure of snake venom CRAMPs was performed by Wang et al. (2008). These authors isolated a potent cathelicidin-like AMP (cathelicidin-BF) from snake venoms of *B. fasciatus* and, by means of biophysical studies (CD and NMR), characterized this peptide's structure. Initially, CD spectra were recorded in hydrophilic, hydrophobic, and anionic environments, suggesting a coil-to-helix transition from water to 2,2,2-trifluroethanol (TFE) and from water to sodium docecyl sulfate micelles. Although CD provides useful information on peptides' and proteins' secondary structure, it does not indicate which residues participate or not in secondary structure stabilization. Therefore, in addition to the CD data for cathelicidin-BF, those authors also performed 2D-NMR experiments, revealing that, in TFE/water mixtures, the α-helical segment in cathelicidin-BF is continuous from Phe^2^ to Phe^18^. However, the extension of this segment is interrupted from Lys^19^ to Phe^30^, which may be explained by the presence of a proline residue at position 21 (Wang et al., 2008). Similar findings were observed for the first CRAMP isolated from sea snakes, named Hc-CATH, which was structurally characterized *in silico* and *in vitro* (Wei et al., 2015). Proline is commonly associated with the limitation of α-helix formation in AMPs, both in aqueous solution and membrane-like conditions, rendering proline-containing AMPs more flexible than proline-free AMPs (Yang et al., 2006). Therefore, cathelicidin-BF was characterized as an α-helical peptide with a flexible C-terminal tail (Wang et al., 2008). Finally, this structural profile (helix-Pro-coil) seems to play a crucial role for the potent antibacterial property of cathelicidin-BF, as its truncated analog (cathelicidin-BF15) did not display promising activities against a range of bacteria (Wang et al., 2008; Chen et al., 2011).

Similarly, de Latour et al. (2010) and Dean et al. (2011) have characterized the secondary structure of NA-CATH, a *N. atra* cathelicidin, through CD experiments in different conditions. As for cathelicidin-BF, NA-CATH has a proline residue at the C-terminal region (position 25). Due to this helix-breaker residue, NA-CATH has shown weaker helical CD signatures than other cathelicidin-like peptides designed based on the 11-residue pattern (KR(F/A)KKFFKK(L/P)K), which is derived from the natural NA-CATH (de Latour et al., 2010). Moreover, these studies have correlated the higher helical propensity of NA-CATH short analogs to their higher antibacterial and antibiofilm activities, when compared to the natural NA-CATH (Dean et al., 2011). These data, along with those for cathelicidin-BF, raise the question of whether the α-helical extension in the natural peptides would lead to improved antimicrobial potential or not, thus shedding some light on the role of C-terminal flexibility in these natural CRAMPs.

More recently, the tridimensional structure of NA-CATH has been determined using NMR (Du et al., 2015). Du et al. (2015) reported that, in the presence of 30% TFE in phosphate-buffered saline, NA-CATH presents a defined α-helix from Phe^3^ to Lys^23^, whereas a random coil conformation is observed due to the presence of Pro^25^. Previous works have shown that NA-CATH causes complete lysis of anionic liposomes and rapidly induces bacterial membrane disruption (Juba et al., 2015). Therefore, NMR and fluorescence re-quenching experiments have been carried out to evaluate the behavior of this peptide in the presence of liposomes (Du et al., 2015). NMR analyses revealed fast exchange in the peptide/liposome complexes, along with signal broadening for aromatic residues, thus indicating their interaction with the liposomes. From these analyses, the authors also concluded that a significant portion of NA-CATH is in solution, which may suggest a compromised liposome bilayer. Furthermore, the C-terminal flexible region of NA-CATH, which is also recurrent in other natural snake venom CRAMPs, is more stable (less mobile) in the presence of liposomes. Finally, Du et al. (2015) also observed that fluorophore leakage suggests that NA-CATH acts on bacteria by either membrane thinning or transient pore formation, corroborating the NMR data (Du et al., 2015).

**Figure 3:**
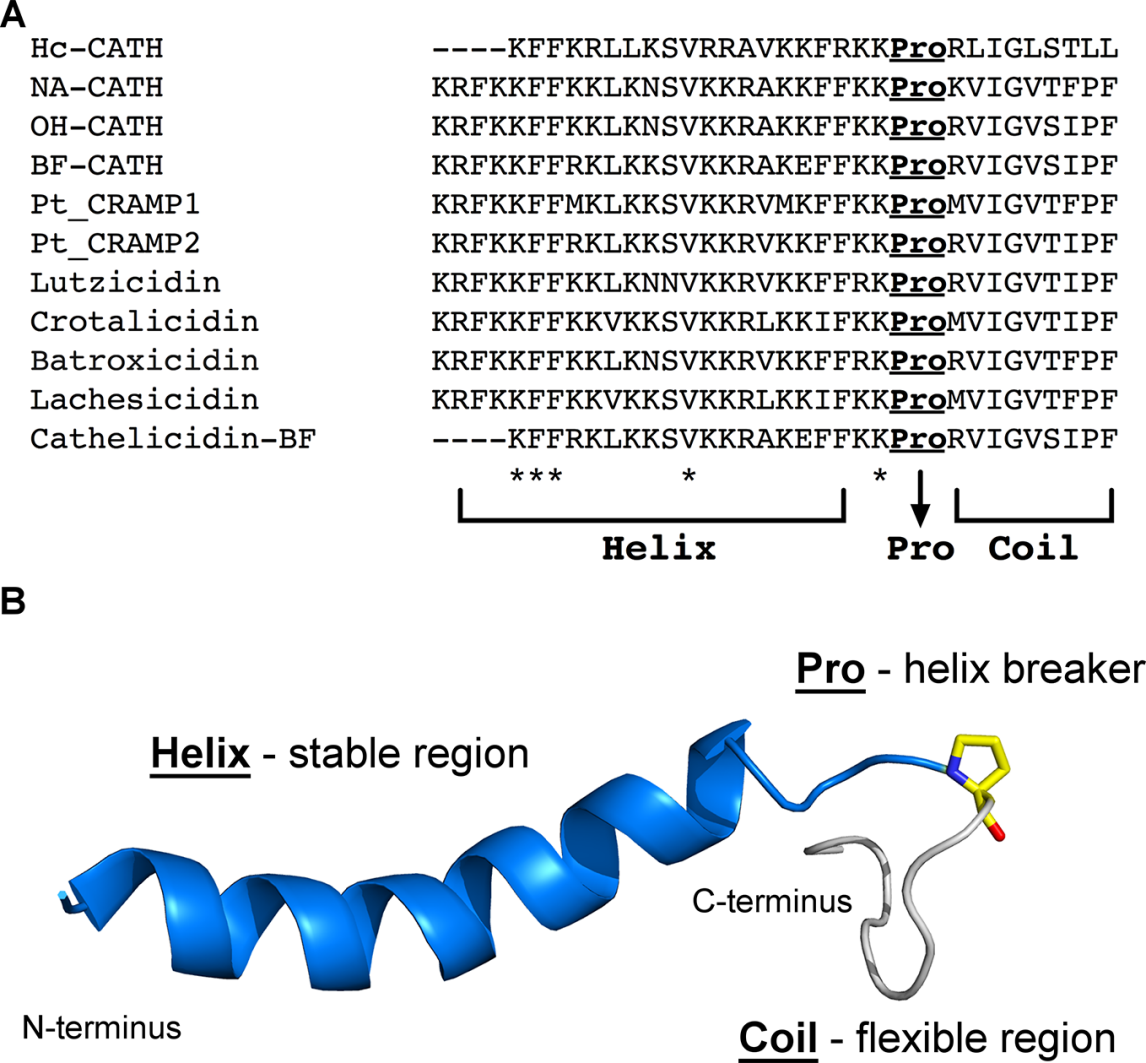
Representation of the helix-Pro-coil structural profile that has been described for snake venom CRAMPs. **(A)**, Sequence alignment highlighting a conserved proline residue at the C-terminus region of all snake venom CRAMPs here described. *Indicates conserved residues between all sequences. **(B)**, Lowest free energy structure obtained by solution NMR for crotalicidin (PDB entry: 2mwt) (Falcao et al., 2015). Although the two lysine residues that precede the proline appear unstructured, they are structurally stable among the 20 lowest free energy structures deposited for crotalicidin. In contrast, the residues after the proline are highly flexible. The proline residue is represented as yellow sticks.

As mentioned above, the structure-function relationship in natural snake venom CRAMPs that adopt a helix-Pro-coil structural profile (**Figure 3**) is still under investigation. Bearing this in mind, Falcao et al. (2015) performed the *in silico* dissection of the natural CRAMP Ctn, resulting in two fragments, Ctn [1–14] and Ctn [15–34]. In parallel to what has been described for cathelicidin-BF and Na-CATH, the full-length Ctn adopts a helix-Pro-coil structure in the membrane-like environment (dodecylphosphocholine micelles). NMR studies also showed that Ctn [1–14] adopts a well-defined amphipathic α-helix, whereas Ctn [15–34] remains unstructured. Interestingly, however, the antibacterial and anticancer properties of natural Ctn were retained solely in the unstructured fragment, Ctn [15–34]. These findings, along with the current literature on AMPs, support the hypothesis that structural flexibility may play a key role in the function of these molecules (Amos et al., 2016; Cardoso et al., 2018a).

### Conclusions and Prospects

Although few or no new classes of antibiotics have reached the pharmaceutical industry for some years, there is a relentless interest in the development of novel antibacterial agents from AMPs for therapeutic uses. Facing this scenario, many studies have been carried out with the purpose of characterizing new bioactive molecules, as well as their mechanisms of action on the target of clinical interest, here potentially found in reptilian peptides.

As reported in this review, CRAMPs derived from snake venoms have been investigated in respect to structure and mechanism of action, relating with their bactericidal and immunomodulatory activity, as promising molecules that could be developed into future antibiotics in clinical therapy. In general, these cathelicidins have demonstrated potent *in vitro* and *in vivo* antimicrobial activity, including against some multidrug-resistant strains. However, more targets can be exploited in view of the wide range of biological functions that natural cathelicidins isolated from snake venom may present.

Moreover, strategies for developing cathelicidin-based antibiotics have been employed to overcome some obstacles regarding AMP translation to the clinic, including particularly low structural stability, biocompatibility, oral bioavailability, size, and cytotoxicity (Mahlapuu et al., 2016; Costa et al., 2019). Thereby, computational studies like the rational design of molecules based on three-dimensional structures have been widely applied to optimize or improve their activity, as well as reducing the costs of production of new drugs (Mishra et al., 2017). Some of the most successful modifications include substitutions, insertions, or deletions of amino acid residues in the primary sequence of natural peptides, which may change their hydrophobicity and hydrophilicity, reduce their cytotoxic effect and/or render them less susceptible to proteolytic degradation (Fjell et al., 2012; Cardoso et al., 2018a; Costa et al., 2019). Furthermore, these modifications allow the design of peptide analogs with a reduced number of amino acid residues, while preserving the biological properties of the parent peptide.

These molecules present a high applicability degree as antimicrobial drugs, especially for multidrug-resistant bacterial infections treatment, one of the major health threats of the 21st century. Although cathelicidins size remains as a restriction for their large-scale production (chemical synthesis), this class of natural peptides has shown great biotechnological and pharmacological potential, thus highlighting their importance as model molecules for future peptide-based therapies (Cardoso et al., 2018a; Costa et al., 2019).

## Author Contributions

EB, RG, MC, NS, OF and EC contributed substantially with data research and discussions of the content; they wrote the article, and reviewed and edited the manuscript before submission.

## Funding

This work was supported by Conselho Nacional de Pesquisa (CNPq); Coordenação de Aperfeiçoamento Pessoal de Nível Superior (CAPES); Fundação de Apoio à Pesquisa do Distrito Federal (FAPDF) and Fundação de Apoio ao Desenvolvimento do Ensino, Ciência e Tecnologia do Estado de Mato Grosso do Sul (FUNDECT), Brazil.

## Conflict of Interest

The authors declare that the research was conducted in the absence of any commercial or financial relationships that could be construed as a potential conflict of interest.
