# Supplementary material for: Snake Venom Cathelicidins as Natural Antimicrobial Peptides
Source: Front Pharmacol. 2019 Nov 29;10:1415. doi: 10.3389/fphar.2019.01415 (PMC6895205; doi:10.3389/fphar.2019.01415)
Supplement: Supplementary file 1 [file DataSheet_1.pdf]

Supplementary Table 1. Reptilian cathelicidins (non-snake) deposited in the Antimicrobial Peptide Database (APD) with listed antibacterial and antibiofilm activities.

**ID and peptide name**	**Organism**	**Active peptide sequence**	**Bacterial strains**	**Activity**		**Reference**
				**Antibacterial and Antibiofilm ($\mu g.mL^{-1}$)**	**Other activities**	
AP03078<br><br>Cm-CATH1	Green sea turtle<br>*Chelonia mydas*	RRSIFRKLRRKIKKGLKKGIQ<br>HLLAGGRQGLPQGGRPGMI	*Acinetobacter baumannii*	>200*	Antifungal	(Qiao et al., 2019)
			Bacillus cereus	75*		
			Bacillus subtilis	18,75*		
			Enterococcus faecalis	>200*		
			Enterococcus faecium	4,69*		
			Escherichia coli	4,69 to >200*		
			Klebsiella oxytoca	>200*		
			Klebsiella pneumoniae	75 to >200*		
			Nocardia asteroides	4,69*		
			Proteus mirabilis	>200*		
			Proteus vulgaris	>200*		

			Pseudomonas aeruginosa	4,69 to >200*		
			Serratia marcescens	>200*		
			Shigella dysenteriae	4,69*		
			Staphylococcus aureus	18,75 to >200*		
			Staphylococcus epidermidis	>200*		
			Stenotrophomonas maltophilia	9,38*		
			Acinetobacter baumannii	18,75*		
			Bacillus cereus	9,38*		
			Bacillus subtilis	4,69*		
			Enterococcus faecalis	75*		
			Enterococcus faecium	2,34*<br>10**<br>25***		
			Escherichia coli	4,69 to 9,38*<br>25***		

AP03079<br>Cm-CATH2		RRSRFGRFFKKVRKQLGRVL<br>RHSRITVGGRMRF	*Klebsiella oxytoca*	18,75*	Antifungal<br>Anti-inflammatory	
			Klebsiella pneumoniae	9,38 to 18,75*		
			Nocardia asteroides	2,34*		
			Proteus mirabilis	18,75*		
			Proteus vulgaris	18,75*		
			Pseudomonas aeruginosa	2,34 to 9,38*		
			Serratia marcescens	>200*		
			Shigella dysenteriae	1,17*		
			Staphylococcus aureus	4,69 to >200*<br>10**<br>25***		
			Staphylococcus epidermidis	9,38*		
			Stenotrophomonas maltophilia	18,75*		
AP03080		TRGRWKRFWRGAGRFFRRH<br>KEKIIRAAVDIVLS	*Acinetobacter baumannii*	>200*	Antifungal	
			Bacillus cereus	9,38*		

Cm-CATH3			*Bacillus subtilis*	1,17*		
			Enterococcus faecalis	37,5*		
			Enterococcus faecium	1,17*		
			Escherichia coli	1,17 to 9,38*		
			Klebsiella oxytoca	>200*		
			Klebsiella pneumoniae	9,38 to 18,75*		
			Nocardia asteroides	2,34*		
			Proteus mirabilis	>200*		
			Proteus vulgaris	>200*		
			Pseudomonas aeruginosa	2,34 to 37,5*		
			Serratia marcescens	>200*		
			Shigella dysenteriae	1,17*		
			Staphylococcus aureus	1,17 to >200*		
			Staphylococcus epidermidis	37,5*		
			Stenotrophomonas	>200*		

			maltophilia			
AP03081<br><br>Cm-CATH4		MAFPFSTQRINPEIEEGNASLA<br>DLPVTHAGSLPGIKAQVRTAL<br>GIALLLVA	*Acinetobacter baumannii*	>200*	Antifungal	
			Bacillus cereus	>200*		
			Bacillus subtilis	9,38*		
			Enterococcus faecalis	>200*		
			Enterococcus faecium	4,69*		
			Escherichia coli	9,38 to >200*		
			Klebsiella oxytoca	>200*		
			Klebsiella pneumoniae	37,5 to >200*		
			Nocardia asteroides	37,5*		
			Proteus mirabilis	>200*		
			Proteus vulgaris	>200*		
			Pseudomonas aeruginosa	>200*		
			Serratia marcescens	>200*		
			Shigella dysenteriae	9,38*		
			Staphylococcus aureus	18,75 to >200*		

			Staphylococcus epidermidis	>200*		
			Stenotrophomonas maltophilia	75		
AP03050 Ps-CATH4	Chinese soft-shelled turtle *Pelodiscus*	TRGRWGRFKRRAGRFIRRNRWQIISTGLKLIG	*Acinetobacter baumannii*	>200*	Antifungal	(Shi et al., 2019)
			Bacillus cereus	2,34*		
			Bacillus subtilis	9,38*		
			Dysentery bacillus	2,34		
			Enterococcus faecalis	>200*		
			Enterococcus faecium	9,38*		
			Escherichia coli	9,38*		
			Klebsiella oxytoca	4,69*		
			Klebsiella pneumoniae	9,38 to 18,75*		
			Nocardia asteroides	9,38*		
			Proteus mirabilis	9,38*		
			Proteus vulgaris	>200*		

	sinensis		*Pseudomonas aeruginosa*	9,38 to 18,75*		
			Salmonella paratyphi	9,38*		
			Serratia marcescens	>200*		
			Staphylococcus aureus	9,38* to >200*		
			Staphylococcus epidermidis	18,75*		
			Stenotrophomonas maltophilia	9,38 to 75*		
			Acinetobacter baumannii	>200*		
			Bacillus cereus	>200*		
			Bacillus subtilis	>200*		
			Dysentery bacillus	>200*		
			Enterococcus faecalis	>200*		
			Enterococcus faecium	>200*		
			Escherichia coli	18,75 to >200*		
			Klebsiella oxytoca	75*		

AP03051<br><br>Ps-CATH6		KKPSKKPKPQAMTFPKVTVE<br>YFPASFSTAALTVPED	*Klebsiella pneumoniae*	37,5 to >200*	Antifungal	
			Nocardia asteroides	>200*		
			Proteus mirabilis	>200*		
			Proteus vulgaris	>200*		
			Pseudomonas aeruginosa	37,5 to >200*		
			Salmonella paratyphi	>200*		
			Serratia marcescens	>200*		
			Staphylococcus aureus	>200*		
			Staphylococcus epidermidis	>200*		
			Stenotrophomonas maltophilia	75 to >200*		
NA<br><br>AM-		GLFKKLRRKIKKGFKKIFKRL	*Acinetobacter baumannii*	10 to 42*	NR	
			Enterococcus faecalis	>42*		
			Escherichia coli	21 to 140*		
			Klebsiella pneumoniae	5,2 to 28*		

CATH21	American alligator *Alligator mississippiensis*		*Pseudomonas aeruginosa*	10 to 42*		(Barksdale et al., 2017)
			Staphylococcus aureus	28		
NA AM-CATH28		KIKKGFKKIFKRLPPIGVGVSIPLAGKR	*Acinetobacter baumannii*	10 to 28*	NR	
			Enterococcus faecalis	>140*		
			Escherichia coli	>140*		
			Klebsiella pneumoniae	5,6 to 28*		
			Pseudomonas aeruginosa	21 to >42*		
			Staphylococcus aureus	>42*		
NA AM-CATH36		GLFKKLRRKIKKGFKKIFKRLPPIGVGVSIPLAGKR	*Acinetobacter baumannii*	5,2*	NR	
			Enterococcus faecalis	>42*		
			Escherichia coli	140*		
			Klebsiella pneumoniae	5,2 to 5,6*		
			Pseudomonas aeruginosa	5,2*		
			Staphylococcus aureus	21*		
			Acinetobacter baumannii	4,69*		
			Bacillus cereus	9,38*		

AP02898<br><br>As-CATH4	Clinese alligator<br>*Alligator sinensis*	RRGLFKKLRRKIKKGFKKIFK<br>RLPPVGVGVSIPLAGRR	*Bacillus subtilis*	2,35*	Antifungal	(Chen et al., 2017)
			Enterococcus faecalis	>200*		
			Enterococcus faecium	9,38*		
			Escherichia coli	9,38 to >200*<br>18,75**<br>40***		
			Klebsiella oxytoca	9,38*		
			Klebsiella pneumoniae	9,38 to 18,75*		
			Nocardia asteroides	>200*		
			Proteus mirabilis	9,38*		
			Proteus vulgaris	9,38*		
			Pseudomonas aeruginosa	9,38 to 18,75*		
			Salmonella paratyphi	4,69*		
			Shigella dysenteriae	9,38*		
			Staphylococcus aureus	9,38 to >200*		
			Staphylococcus epidermidis	9,38*		

			Stenotrophomonas maltophilia	18,75 to 37,5*		
AP02899 As-CATH5		TRRKFWKKVLNGALKIAPFLLG	*Acinetobacter baumannii*	18,75 to 200*	Antifungal	
			Bacillus cereus	18,75*		
			Bacillus subtilis	2,35*		
			Enterococcus faecalis	9,38*		
			Enterococcus faecium	18,75*		
			Escherichia coli	9,38 to 18,75*<br>18,75**<br>40***		
			Klebsiella oxytoca	938*		
			Klebsiella pneumoniae	9,38 to >200*		
			Nocardia asteroides	9,38*		
			Proteus mirabilis	4,69*		
			Proteus vulgaris	>200*		
			Pseudomonas aeruginosa	9,38 to 18,75*		
			Salmonella paratyphi	9,38*		

			Shigella dysenteriae	9,38*		
			Staphylococcus aureus	9,38 to 37,5*		
			Staphylococcus epidermidis	18,75*		
			Stenotrophomonas maltophilia	9,38*		
AP02900		TRWLWLLRGGLKAAGWGIR	*Acinetobacter baumannii*	>200*	Antifungal	
			Bacillus cereus	>200*		
			Bacillus subtilis	9,38*		
			Enterococcus faecalis	>200*		
			Enterococcus faecium	>200*		
			Escherichia coli	4,69 to 18,75*<br>18,75**<br>40***		
			Klebsiella oxytoca	9,38*		
			Klebsiella pneumoniae	9,38*		
			Nocardia asteroides	18,75*		

As-CATH6		AHLNRNQ	*Proteus mirabilis*	9,38*		
			Proteus vulgaris	>200*		
			Pseudomonas aeruginosa	18,75 to 37,5*		
			Salmonella paratyphi	37,5*		
			Shigella dysenteriae	37,5*		
			Staphylococcus aureus	9,38 to >200*		
			Staphylococcus epidermidis	>200*		
			Stenotrophomonas maltophilia	18,75 to >200*		

Abbreviations: *MIC (minimal inhibitory concentration); **Inhibition of biofilm formation; ***Eradication of preformed biofilms; NA= Not available; NR= Not rated
